# Hepatocellular Carcinoma in Patients with a Sustained Response to Anti-Hepatitis C Therapy

**DOI:** 10.3390/ijms160819698

**Published:** 2015-08-19

**Authors:** Roberta D’Ambrosio, Cristina Della Corte, Massimo Colombo

**Affiliations:** Division of Gastroenterology and Hepatology, IRCCS Ca’ Granda Ospedale Maggiore Policlinico, Università degli Studi di Milano, Milan 20122, Italy; E-Mails: cristina_dellacorte@hotmail.it (D.C.C.); massimo.colombo@unimi.it (C.M.)

**Keywords:** hepatitis C, hepatocellular carcinoma, interferon, sustained virological response

## Abstract

Hepatocellular carcinoma (HCC) is a common, life-threatening complication of longstanding infection with the hepatitis C virus (HCV), likely a consequence of the direct oncogenic activity of the virus cooperating with liver cell inflammation in transforming the liver into a mitogenic and mutagenic environment. The achievement of a sustained virological response (SVR) to interferon-based therapies has been shown to benefit the course of hepatitis C in terms of reduced rates of liver-related complications and mortality from all causes. Interestingly, while achievement of an SVR is associated with a negligible risk of developing clinical decompensation over the years, the risk of HCC is not fully abrogated following HCV clearance, but it remains the dominant complication in all SVR populations. The factors accounting for such a residual risk of HCC in SVR patients are not fully understood, yet the persistence of the subverted architecture of the liver, diabetes and alcohol abuse are likely culprits. In the end, the risk of developing an HCC in SVR patients is attenuated by 75% compared to non-responders or untreated patients, whereas responders who develop an HCC may be stratified in different categories of HCC risk by a score based on the same demographic and liver disease-based variables, such as those that predict liver cancer in viremic patients. All in all, this prevents full understanding of those factors that drive HCC risk once HCV has been eradicated. Here, we critically review current understanding of HCC in SVR patients focusing on factors that predict residual risk of HCC among these patients and providing a glimpse of the expected benefits of new anti-HCV regimens based on direct antiviral agents.

## 1. Introduction

Chronic infection with the hepatitis C virus (HCV) is a leading cause of liver-related mortality worldwide, since it is a major risk factor for the development of cirrhosis and hepatocellular carcinoma (HCC) [1]. Antiviral therapy with interferon (IFN) has long been the only available option to modify the course of the infection, since in many patients with a sustained virological response (SVR), it succeeded in preventing the onset of life-threatening end-stage complications of hepatitis C [2,3,4,5,6,7]. With all of the caveats related to the retrospective design and inherent selection biases, IFN studies associated achievement of an SVR with a 75% reduction of the incidence of HCC in patients who were treated at various histological stages of infection [8]. Owing to the residual risk of HCC, which has been estimated to be around 0.5%–1% per year, cirrhotic patients successfully achieving an SVR are recommended to continue surveillance with abdominal ultrasound (US) in order to detect HCC early and improve treatment outcome [9]. In the face of the residual incidence of such a life-threatening complication of HCV as HCC, a few studies have only searched for factors responsible for the increased risk of this tumor in SVR patients. With the advent of user-friendly, highly effective and safe IFN-free therapies based on the combination of direct antiviral agents (DAA), a greater impact of antiviral therapy on the prevention of HCV-related complications is awaited, particularly in patients with advanced cirrhosis and those with decompensated liver disease who are unfit for IFN therapy, while they are at increased risk of HCC development.

Here, we review the oncogenic mechanisms of HCV and how HCC risk is modified by successful anti-HCV therapy, focusing on the role of fibrosis regression and risk factors for HCC.

## 2. HCC in HCV-Infected Patients

HCC is an inflammatory-type neoplasia, which results from the interaction between direct carcinogenic effects of HCV proteins, which deregulate host cell cycle check points, and the immune and virus-mediated oxidative stress, causing DNA mutations in both infected and uninfected liver cells [10]. From a clinical point of view, neoplastic transformation of uninfected liver cells under the pressure of inflammatory stimuli released by HCV-infected hepatocytes is of strategic importance in the understanding of HCC, which develops years after treatment-induced eradication of infection, as well as of tumors recurring in SVR patients years after a successful ablation of an HCC. While advanced fibrosis stands as a relevant determinant of HCC risk in both HCV viremic and SVR patients, patient age, alcohol abuse and insulin resistance appear to be associated with an increased risk of HCC in both patients categories, supporting a multifactorial origin of this neoplasia. Despite initially promising results, more than one study investigating genetic predisposition to liver cancer failed to identify any robust predictor of HCC development in HCV patients that can be used in clinical practice to optimize the management of patients with a liver cancer. More recently, the PNPLA3 polymorphism rs738409 has been identified to exert a marked influence on hepatocarcinogenesis in patients with cirrhosis of European descent; however, these data need further validation. In fact, most publications suffer from major methodological drawbacks because of their case-control, retrospective and single-center design, mainly involving selected Asian populations. Prospective cohort studies conducted in large homogeneous populations with a sufficient number of events during follow-up require a long time to be conducted and, therefore, are still scarce [11].

## 3. The Clinical Benefits of an SVR to Interferon

In patients with advanced liver fibrosis due to HCV, achievement of an SVR has been associated with a significant reduction of such life-threatening complications as liver failure and HCC [2,3,4,5,6,7]. Owing to the fact that clinical decompensation rarely occurs in SVR patients, HCC stands as the commoner complication in patients who are successfully treated with IFN-based regimens, with a reported incidence of less than 1% [2,3,4,5,6,7,12,13] (Table 1). While the persistence of HCC in SVR patients might well reflect the pro-carcinogenic effects of residual cirrhosis, alternatively, data suggest a pathogenetic role of non-virus-related carcinogenic factors, like diabetes and alcohol, as well as the confounding effect of time required for newly-developing HCC to become clinically apparent [14].

Clinical benefits following HCV eradication were first reported in a retrospective analysis of 329 compensated cirrhotics in Europe, who were followed-up for 45 (6–93) months after the end of IFN therapy [15]. That study reported no events among 14 responders, compared to a five-year estimated risk of 2.1% of HCC and 7% of decompensation in untreated patients, respectively. These preliminary observations were confirmed and strengthened by a study in Japan, which added evidence of improved survival in SVR patients where HCC, which was detected in less SVR patients (27/836) than in non-responders (214/1556) or untreated (67/395) cirrhotics, was the dominant cause of liver-related mortality in patients not achieving an SVR (66%) [2]. These were also the findings of a retrospective multicenter study in Italy involving 1214 patients with cirrhosis and 124 SVR patients who were followed for 96 (6–167) months [4]. In that study, HCC was the only complication occurring among patients with an SVR (SVR *vs.* non-SVR, 5.6% *vs.* 16%, *p* < 0.001), whereas no other liver-related events were registered. Although not linear, the yearly incidence rate of HCC in SVR cirrhotics was lower than in non-responders (0.66 *vs.* 2.10), the latter ones having a 2.59-fold higher risk of developing an HCC. While older age (>54 years), male gender, low platelet count and absence of SVR were independently associated with an increased risk of developing liver cancer, liver-related mortality was higher in non-SVR than in SVR patients (RR 7.59 (1.84–31.29), *p* < 0.01), all-cause mortality rates, however, remaining unaffected [4]. Likewise, Veldt and colleagues [5] reported lower HCC rates among 142 SVR patients with a histological pre-treatment diagnosis of advanced fibrosis (Ishak score S4–S6), which however did not exceed the rates reported in non-responders at five years (*p* = 0.192). Moreover, in that study, HCC developed between 1.7 and 3.9 years from treatment failure, with an incidence of 1.8% at one year and 9.2% at five years, *i.e*., at higher rates than those observed in the Italian study. This was also the message of the scrutiny of 307 HCV patients with advanced liver disease in France who, after being treated with IFN-based therapies, were regularly followed-up for 3.5 (1–18) years [16]. HCC developed in six out of the 103 patients with an SVR (5.8%), with an estimated yearly incidence rate of 1.24 (95% C.I. 0.28–2.20), which was lower than the rates observed among non-responders (20%), with an annual incidence rate of 5.85 (95% C.I. 4.23–7.47). In this study, too, treatment failure was an independent predictor of HCC risk, together with older age (>60 years) and such markers of hepatitis severity as high bilirubin values, low platelet count and low serum albumin. The existence of a relationship between treatment failure and HCC risk in patients with advanced HCV emerged also in a multinational study of 530 patients who were followed-up for a median of 8.4 (6.4–11.4) years after treatment completion: an SVR was achieved by 192 (36%), seven of whom (4%) developed HCC during seven years from treatment completion, *i.e*., at a lower rate than that reported in non-SVR patients (18%) [7]. Despite intense scrutiny of the databases, all of these studies failed to identify predictors of increased HCC risk in SVR patients other than those predicting HCC in viremic patients. All in all, these retrospective studies validated the findings of the only prospective investigation with IFN that was carried out in Japan in 271 patients who were followed-up for seven years after treatment completion [17]. In that cohort, the rates of HCC and liver-related death were lower in SVR patients than in non-responders (11/64 *vs.* 73/207, 17% *vs.* 35%, *p* = 0.008 and 0/64 *vs.* 32/207, 0% *vs.* 15%, *p* = 0.0002), whereas rates of HCC were also lower in SVR patients when compared to untreated patients (age-adjusted hazard ratio 0.31 (95% C.I., 0.16–0.61), *p* < 0.001). Importantly, none of the SVR patients died of liver cancer. It should be mentioned, however, that assessment of clinical benefits provided by an SVR to IFN may be biased by methodological flaws in the design and conduct of studies that were originally designed to evaluate the antiviral activity of IFN, not its anticancer properties. In those studies, in fact, patient enrollment was skewed toward highly-selected individuals who were fit to IFN therapies, thereby excluding a majority of patients with more advanced liver disease who were IFN unable or intolerant, while notoriously being at high risk of developing HCC. Further, none of these studies had patients stratified pretreatment for relevant predictors of HCC risk, like age, hepatitis severity, alpha-fetoprotein (AFP) levels and co-morbidities, therefore making a comparison of the outcomes between treated and untreated patients difficult. Finally, the length of and adherence to follow-up between treated and untreated patients was substantially heterogeneous, a fact that, together with the lack of a predefined strategy of HCC surveillance, led to inaccurate estimates of HCC rates in most studies.

## 4. Expected Benefits of All Oral Anti-HCV Therapy

All oral therapy of hepatitis C is expected to provide additional clinical benefits with respect to IFN-based regimens [18]. Second wave direct antiviral agents, in fact, are virtually applicable to all patients with HCV, independently of the status of clinical compensation and the presence of comorbidities, and are safe and highly effective against all genotypes of HCV, while being generally well tolerated. Combination therapies based on an NS5A inhibitor associated with other classes of DAA guarantee more than 95% SVR rates in patients with chronic hepatitis C due to the difficult to cure genotype 1 and more than 90% rates in those with compensated cirrhosis, increasing by 2.6-fold the success rate of antiviral therapy in terms of intention to treat analysis compared to IFN-based regimens [19,20]. Importantly, oral regimens were highly effective and safe in patients with decompensated cirrhosis who are interferon unable, a fact that expand the spectrum of clinical benefits of antiviral therapy in the hepatitis C scenario [19,20,21]. While an SVR in patients with cirrhosis is definitely associated with high rates of liver function improvement in terms of both MELD and Child Pugh scores, what is still unclear is whether the risk of developing a liver cancer in patients treated with oral regimens is attenuated compared to what has been seen in patients treated with IFN. Translating our experience to patients with chronic hepatitis B, in whom the introduction of highly effective and safe NUC analogs to treat HBV expanded survival expectancy favoring the onset of liver cancer in survivors [22,23], one wonders whether a similar scenario can be foreseen in HCV. A preliminary report in 120 patients with advanced cirrhosis who received a 12-week course of Sofosbuvir (a nucleotide inhibitor of NS5B) + Simeprevir (a NS3-4A protease inhibitor) provided an overall rate of 81% SVR, but left a substantial number of patients at risk of developing HCC in the 12 months post-treatment.

**Table 1:** Estimated annual incidence and associated risk factors of hepatocellular carcinoma (HCC) in HCV patients with cirrhosis or advanced fibrosis stratified by treatment response. SVR, sustained virological response.

Reference	No. of Patients	Follow-up Months (range)	HCC Risk Factors	HCC Incidence	
				SVR +	SVR −
Yoshida *et al*., 2004 [2]	2392 (7)	45 (6–93)	** Advanced fibrosis; age > 60 male sex	0.4%	1.7%
Shiratori *et al*., 2005 [17]	271 (7)	100.8 (76.8–136.8)	Age > 60 albumin < 4 mg/dL	2.4%	5%
Bruno *et al*., 2007 [4]	920 (8)	96 (6–169)	Cirrhosis; age > 54 Male sex; platelets < 109,000	0.7%	2%
Cardoso *et al*., 2010 [16]	307 (18)	42 (12–216)	Cirrhosis; bilirubin > 0.9 mg/dL albumin < 4 g/dL platelets < 150,000	0.3%	1.1%
Yu *et al*., 2006 [3]	1619 (16)	70 (12–180)	Genotype 1; age	0.76%	2.2%
Veldt *et al*., 2007 [5]	479 (4.9)	25.2 (9.6–58.8)	No features found	0.4%	1.9%
Van der Meer, 2012 [7]	530 (11.4)	100 (77–144)	Male sex; age > 49 diabetes genotype 3; alcohol abuse	0.3%	1.6%
Mallet *et al*., 2008 [6]	96 (11.5)	118 (86–138)	* Histological cirrhosis persistence; anti-Hbc +	0.3%	1.3%
Fattovich *et al*., 1997 [15]	329 (12.7)	60	Bilirubin > 1 mg/dL age > 57	1%	2.3%

* SVR patients; ** non-SVR and untreated patients.

## 5. Indicators of HCC Risk in SVR Patients

Owing to the low incidence of HCC among SVR patients, the study of factors associated with liver cancer development in this population is rather problematic. So far, no single clinical or histological predictor of HCC development has been identified in SVR patients, reinforcing therefore the concept that HCC risk in HCV patients may be multifactorial [2,3,4,5,6,17].

### 5.1. Host-Related Predictors

In 2012, Chang and colleagues developed a predictive score (Score_HCC_) for HCC development in patients with an SVR following the scrutiny of more than 800 SVR patients who were followed-up for 41.3 (3.5–113.9) months after anti-HCV treatment completion.(22) As older age (>65 years), advanced fibrosis (F3–F4), lower platelets count (<150,000/mm^3^) and high AFP levels (≥20 ng/mL) were found to be independent risk factors for HCC, a predictive model was constructed attributing points to these variables, which allowed stratification of patients into three risk groups for HCC: low risk (Score_HCC_ ≤ 10 points), intermediate risk (Score_HCC_ 11–15) and high risk (Score_HCC_ ≥ 16). The risk of HCC, in fact, progressively increased across groups from 1.37%–9.14% and 30.77% (*p* < 0.001), but among the 37 SVR patients who developed HCC during a median follow-up of four years, more than half were in the low-risk category, whereas, as expected, the incidence of HCC gradually increased over post-treatment follow up. While all of these findings stress the importance of time factors in HCC development, they actually work against the application of this propensity score with the aim of optimizing screening strategies in the hyperendemic areas of hepatitis, mainly owing to the many patients in the lower bound of risk who in the end developed a liver cancer. The study was also far from being accurate in the search of HCC predictors, as it failed to provide information on the potential role of such relevant exogenous cofactors as alcohol and BMI that potentially might have tuned the residual risk of liver cancer in many patients [24]. This notwithstanding, if validated, this HCC score algorithm might be considered for improving the design of studies of HCC prevention, whereas it does not fit the safety criteria for refining strategies of surveillance in terms of cost effectiveness. This algorithm has not been validated in the West: in a multinational study in the U.S., Canada and Europe, age at the time of antiviral treatment emerged as the only variable associated with an increased risk of HCC [25]. Though rates of HCC were 2.6% for patients <45 years, 9.3% for patients 45–60 years and 12.2% for patients >60 years (*p* = 0.006), the study missed data on potential confounders, like the interaction of age with disease severity, diabetes and alcohol. More recently, in a cohort of 642 SVR Asian patients (13% cirrhotics), HCC was strongly associated with cirrhosis (HR 4.98 (95% C.I. 2.32–10.71), *p* < 0.001) and less strongly with age (HR 1.06 (95% C.I. 1.02–1.11), *p* = 0.005) and γGT (HR 1.01 (95% C.I. 1.00–1.013), *p* < 0.001) [26]. Whilst no HCC-associated factors were found among SVR cirrhotics, in non-cirrhotic SVR patients, high baseline γGT (HR 6.44 (95% C.I. 2.20–18.89), *p* = 0.001) and age >60 years (HR 3.68 (95% C.I. 1.33–10.17), *p* = 0.012) were associated with an increased risk of HCC. Interestingly, following stratification of non-cirrhotic SVR patients into three categories at different HCC risk, compared to patients without any risk factor, the HR of HCC was 9.06 (C.I. 2.1–40.9, *p* = 0.004) in patients with one risk factor and 20.62 (C.I. 3.8–112.8, *p* < 0.001) in patients with two risk factors, corresponding to a yearly HCC incidence of 0.14% *vs.* 1.22% *vs.* 4.54% [26].

### 5.2. Residual Liver Fibrosis as a Risk Factor of HCC

A study in France first demonstrated that achievement of an SVR was not enough to prevent cirrhosis-related complications in patients in whom liver fibrosis had not regressed [6]. In that study, 96 HCV patients with a pre-treatment histological diagnosis of cirrhosis, who were treated with IFN-based regimens and followed-up to 118 months after treatment completion, underwent a post-treatment liver biopsy, independently of the treatment outcome, after a median period of 17 months from the end of antiviral therapy. While cirrhosis regression was demonstrated in 18 (19%) patients, 17 with an SVR, liver-related complications occurred less frequently among SVR than among non-responders (10% *vs.* 40%, 11% *vs.* 38%, *p* = 0.009). HCC was the commonest complication occurring in three (8.6%) SVR patients *vs.* 14 (23%) non-responders, whereas all but one SVR patients remained free from decompensation throughout the follow-up period. Interestingly, none of the patients in whom cirrhosis regresses at post-SVR liver biopsy showed any complication, whereas clinical events occurred only among those who were persistently cirrhotic at follow-up biopsy. Among patients with cirrhosis regression, one non-liver-related death was recorded, only. In a study in Italy, regression of cirrhosis was demonstrated in 60% of patients who were examined with a percutaneous liver biopsy performed five years after achieving an SVR, on average [27].

### 5.3. Direct Mechanisms of HCV Carcinogenicity

Occult infection with HCV has long been questioned to explain both hepatic and extrahepatic complications in HCV patients, yet qPCR investigations in various tissues have conclusively demonstrated this hypothesis to be unlikely [28]. Instead, data point to a direct effect of the virus in infected cells that may take place long before treatment-related eradication of the infection, thereby explaining cases of HCC developing years after the curing of hepatitis with IFN. The virus infection results in deregulation of host cell-cycle checkpoints, immune and host-mediated oxidative stress and DNA damage, which may in fact lead to the accumulation of mutations of host DNA, eventually resulting in malignant transformation of infected cells that may require a lengthy time to become clinically evident [10]. The non-structural protein 5B (NS5B)-mediated loss of retinoblastoma protein (Rb) expression likely renders infected hepatocytes unable to mount a normal response to DNA damage and can be expected to promote genomic instability and increase the risk of HCC, while the apparent preservation of miR-122 expression in HCV-associated HCC, despite its loss in HCC due to other etiologies, may account for cancer development within HCV-infected hepatocytes. On the other hand, cells maintaining miR-122 expression would be at risk for persisting direct effects of HCV, being selected during progression toward cancer [10].

### 5.4. Exogenous Risk Factors 

It is well established that many cases of HCC develop in the context of non-virological, environmental risk factors, including alcohol abuse and tobacco smoking [29,30], which may play a role in the residual risk.

Oxidative stress may also be a driving mechanism in HCC developing in alcohol abusers, though a HCC risk threshold for ethanol consumption has not been identified: daily intake of ≥80 g of ethanol for >10 years is thought to increase the risk of HCC by approximately five-fold, women being more vulnerable to alcohol toxicity than men [31,32,33,34,35]. This was the clear message of one study in U.K. where 1.3 million women were involved in breast cancer screening programs, where minor amounts of alcohol consumption, like 10 g/day on average, were associated with a significant increase of HCC risk compared to the general population (increase of RR 24% (95% C.I. 2–51), *p* = 0.03) [35]. Hepatic metabolism of ethanol might lead to increased conversion in the liver of pro-carcinogens into active carcinogens, whereas acetaldehyde and oxygen-free radicals generated by ethanol metabolism may directly harm liver cells by initiating peroxidation of membrane lipids [34]. All in all, oxidative stress is the leading mechanism that transforms the liver in a mitogenic and mutagenic environment [36]. In HCV-infected patients, alcohol may synergically lead to an acceleration of fibrosis deposition and progression to cirrhosis and liver-related complications [37]. The fibrogenetic process induced by alcohol may be accelerated also in patients after clearance of HCV, since residual fibrosis from viral hepatitis may potentially enhance ethanol’s effects. Not surprisingly, therefore, alcohol intake has been associated with HCC development in patients who achieved an SVR to IFN-based therapies, as well. This is the message of a study of 792 SVR patients (1.8% cirrhotics), who were followed-up for 62 months, where at multivariate analysis, alcohol consumption emerged as an independent predictor of HCC (≥50 *vs.* <50 g/day: RR 3.86 (1.58–9.44)), together with F3/F4 fibrosis (RR 5.37 (2.27–12.75)) and older age (3.99 (1.71–9.28)) [38]. Similar results come from another study in Japan, where HCC risk was higher in patients with an alcohol intake ≥27 g/day when compared to non-drinkers (*p* = 0.015), whilst no differences were observed when the threshold of alcohol consumption was raised to ≥80 *vs.* <80 g/day (*p* = 0.447) [39]. More recently, in a cohort of 4302 HCV Japanese patients treated with IFN who were followed up for 8.1 years, a cumulative alcohol intake >200 kg (*p* < 0.05) was also associated with increased risk of HCC following an SVR [40].

There is convincing evidence that both obesity and diabetes may enhance the risk of HCC, independently of a successful antiviral treatment. The importance of diabetes has been confirmed in a recent SEER (surveillance epidemiology and end results)-based re-analysis, showing an up to three-fold increase in the risk of HCC, regardless of the presence of other major risk factors [41]. Further evidence that obesity and diabetes are either jointly or independently associated with an increased risk of HCC is provided by an Italian case-control study and by several large-scale epidemiological studies that have associated the overweight and obesity pandemic in the general population with an increased risk of HCC [42,43]. In a cohort of 900,000 American adults, the risk of dying from liver cancer was in fact 4.5-times higher in men with a BMI ≥ 35 kg/m^2^, compared to men with a normal BMI (18.5 to 24.9 kg/m^2^) [29]. In a prospective observational study in Taiwan, extreme obesity (*i.e*., body mass index > 30 kg/m^2^) was independently associated with a four-fold risk of HCC (RR 4.13 (95% C.I. 1.38–12.4)) among anti-HCV positive subjects and a two-fold risk (RR 2.36 (95% C.I. 0.91–6.17)) in those who tested seronegative, thus confirming its potential role in promoting HCC development in SVR patients (RR 1.36 (95% C.I. 0.64–2.89)) [44] These and other studies contributed to the increased recognition of nonalcoholiceatohepatitis (NASH) being a significant cause of both cirrhosis and HCC, with many patients, however, progressing to liver cancer without histological evidence of advanced fibrosis or cirrhosis [45]. Recently, Dyson and colleagues found that nonalcoholic fatty liver disease (NAFLD) accounted for one third of all cases of HCC seen in a referral center in the U.K., whilst in the same period, metabolic risk factors, like hypertension, hypertriglyceridemia, reduced HDL cholesterol and previous cardiovascular events, were present in 66.1% of the patients with a new diagnosis of HCC, irrespective of liver disease etiology [46]. In the last 10 years, evidence has accumulated that HCC in histologically-proven NAFLD often arises without cirrhosis, suggesting that non-cirrhotic HCC may occur more commonly in NAFLD than in liver diseases of other etiologies, whereas HCC has also been reported in patients with metabolic syndrome lacking any histological feature of steatohepatitis and fibrosis [31,47]. The precise mechanisms through which metabolic factors drive HCC development are complex and beyond the purpose of this article; however, major systemic and liver-specific molecular mechanisms, like insulin resistance, hyperinsulinemia, increased expression of tumor necrosis factor signaling pathways and direct lipotoxicity, are major players in the development of HCC [31,47].

Diabetes has long been recognized as a predictor of HCC in patients with chronic hepatitis C (*p* = 0.005), HCC rates decreasing in patients with a mean hemoglobin A1c (HbA1c) level <7.0% during follow-up with respect to patients with an unbalanced diabetes (hazard ratio, 0.56, 95% confidence interval, 0.33–0.89, *p* = 0.015) [40].

Hepatic steatosis after SVR behaves differently from that in NASH, suggesting the possibility that steatosis and HCV infection may cooperate in the development of HCC. In one study, moderate steatosis was an independent risk factor for HCC among 266 patients 10 (±4) years after achieving a SVR, as it occurred in seven (2.6%) patients, only [48]. While pre-treatment histological steatosis (G2-3) emerged as an independent risk factor for HCC (*p* = 0.0002) together with fibrosis stage (F3–F4, *p* = 0.0028) and older age (≥55 years), it declined at the time of HCC diagnosis compared to baseline histological assessment (G0 in one, G1 in three and G2 in one), and interestingly, none of the patients with G0 steatosis at baseline developed an HCC [48].

### 5.5. Tobacco Smoking

While tobacco smoke contains more than one hundred potential carcinogens that may affect the liver, it is still unclear whether tobacco smoking is causally associated with liver cancer [49,50]. A meta-analysis on the effect of smoking on liver cancer [51] reported an overall OR of 1.56 (95% C.I. 1.29–1.87) by comparing current smokers to never smokers and of 1.49 (95% C.I. 1.06–2.10) comparing former smokers to never smokers. The associations among current smokers appeared to be consistent with the overall RR regardless of region, study design, study sample size and publication period. Instead, the synergistic interaction between tobacco smoking and viral hepatitis are inconsistent: a study in Taiwan found a higher RR of tobacco smoking among HBV-negative than among HBV-positive subjects [52], whereas a study in Japan found a higher RR among HBV-positive subjects only [53]. More convincing is the evidence that tobacco smoking is a cofactor for the development of liver cancer in patients with established cirrhosis. These are the conclusions of a retrospective study in China, assessing the smoking habits of 36,000 adults who had died from liver cancer and 17,000 who had died from cirrhosis, showing that among men with chronic HBV infection, HCC risk was 33% in smokers and 25% in nonsmokers [54]. While the RR was independent of age, it was similar in urban and rural areas, was not significantly related to the age when smoking started, but was significantly (*p* < 0.001) greater for cigarette smokers than for smokers of other types of tobacco, with a greater hazard ratio among those who smoked 20/day (for men RR 1.50, 95% C.I. 1.39–1.62, for women RR 1.17, 95% C.I. 1.06–1.29) than among those who smoked fewer cigarettes [54]. Finally, the association between tobacco smoking and HCC risk was also investigated through a case-control study in Italy. Current smoking was unrelated to HCC risk among uninfected individuals (OR 1.0; 95% C.I. 0.5–2.0), but it seemed to enhance the adverse effect of hepatitis viruses among HBsAg+ or anti-HCV+ individuals, with an OR of 23.4 among never or former smokers (at least 12 months) *vs.* 44.3 among current smokers (at least one cigarette/day for at least one year) [55]. The role of tobacco as a cause of residual HCC risk in SVR patients has never been evaluated.

## 6. Conclusions

There is overwhelming evidence that HCV patients who achieve an SVR to interferon-based therapies have a significantly reduced risk of developing a liver cancer in the short/medium term. Additional studies are therefore required to establish whether prevention of HCC in SVR patients is maintained lifelong. The puzzling question of why one quarter of patients who successfully responded to IFN therapy did develop a liver cancer during follow-up remains elusive. HCC risk in fact may be multifactorial, as suggested by its association with patient age and liver disease severity at treatment onset, post-treatment persistence of excessive fibrosis in the liver, coexistence of diabetes, being overweight and alcohol abuse. A worrisome aspect of these findings is the increased HCC risk in older patients and in those with more advanced liver disease, suggesting that long standing infection with this potentially carcinogenetic virus is a primary pathogenetic factor for liver cancer during chronic infection with HCV. This questions current strategies of prioritization of all oral therapy in patients with advanced hepatitis C, which are in place in most European and U.S. States, whereas a refinement of our current policies of HCV therapy worldwide should be pursued. Our strategies against HCC in HCV patients need to be implemented following the finding that metabolic syndrome and alcohol abuse are involved in residual HCC risk in SVR patients. Studies are deemed necessary to establish whether treatment of comorbidities may further contribute to prevention of HCC in HCV patients undergoing successful eradication of the infection.

## References

[1] European Association for Study of Liver (2014). EASL Clinical Practice Guidelines: Management of hepatitis C virus infection. J. Hepatol..

[2] Yoshida H., Tateishi R., Arakawa Y., Sata M., Fujiyama S., Nishiguchi S., Ishibashi H., Yamada G., Yokosuka O., Shiratori Y. (2004). Benefit of interferon therapy in hepatocellular carcinoma prevention for individual patients with chronic hepatitis C. Gut.

[3] Yu M.L., Lin S.M., Chuang W.L., Dai C.Y., Wang J.H., Lu S.N., Sheen I.S., Chang W.Y., Lee C.M., Liaw Y.F. (2006). A sustained virological response to interferon or interferon/ribavirin reduces hepatocellular carcinoma and improves survival in chronic hepatitis C: A nationwide, multicentre study in Taiwan. Antivir. Ther..

[4] Bruno S., Stroffolini T., Colombo M., Bollani S., Benvegnù L., Mazzella G., Ascione A., Santantonio T., Piccinino F., Andreone F., Italian Association of the Study of the Liver Disease (AISF) (2007). Sustained virological response to interferon-alpha is associated with improved outcome in HCV-related cirrhosis: A retrospective study. Hepatology.

[5] Veldt B.J., Heathcote E.J., Wedemeyer H., Reichen J., Hofmann W.P., Zeuzem S., Manns M.P., Hansen B.E., Schalm S.W., Janssen H.L. (2007). Sustained virologic response and clinical outcomes in patients with chronic hepatitis C and advanced fibrosis. Ann. Intern. Med..

[6] Mallet V., Gilgenkrantz H., Serpaggi J., Verkarre V., Vallet-Pichard A., Fontaine H., Pol S. (2008). Brief communication: The relationship of regression of cirrhosis to outcome in chronic hepatitis C. Ann. Intern. Med..

[7] Van der M., Veldt B.J., Feld J.J., Wedemeyer H., Dufour J.F., Lammert F., Duarte-Rojo A., Heathcote E.J., Manns M.P., Kuske L. (2012). Association between sustained virological response and all-cause mortality among patients with chronic hepatitis C and advanced hepatic fibrosis. JAMA.

[8] Morgan T.R., Ghany M.G., Kim H.Y., Snow K.K., Shiffman M.L., de Santo J.L., Lee W.M., di Bisceglie A.M., Bonkovsky H.L., HALT-C Trial Group (2010). Outcome of sustained virological responders with histologically advanced chronic hepatitis C. Hepatology.

[9] For Research, European Organization, and European Association for the Study of the Liver (2012). EASL–EORTC Clinical Practice Guidelines: Management of hepatocellular carcinoma. J. Hepatol..

[10] Lemon S.M. (2012). Is hepatitis C virus carcinogenic?. Gastroenterology.

[11] Trépo E., Nahon P., Bontempi G., Valenti L., Falleti E., Nischalke H.D., Hamza S., Corradini S.G., Burza M.A., Guyot E. (2014). Association between the PNPLA3 (rs738409 C>G) variant and hepatocellular carcinoma: Evidence from a meta-analysis of individual participant data. Hepatology.

[12] Singal A.G., Volk M.L., Jensen D., di Bisceglie A.M., Schoenfeld P.S. (2010). Sustained viral response is associated with reduced liver-related morbidity and mortality in patients with hepatitis C virus. Clin. Gastroenterol. Hepatol..

[13] Aleman S., Rahbin N., Weiland O., Davidsdottir L., Hedenstierna M., Rose N., Verbaan H., Stål P., Carlsson T., Norrgren H. (2013). A risk for hepatocellular carcinoma persists long-term after sustained virologic response in patients with hepatitis C-associated liver cirrhosis. Clin. Infect. Dis..

[14] Lok A.S., Everhart J.E., Wright E.C., di Bisceglie A.M., Kim H.Y., Sterling R.K., Everson G.T., Lindsay K.L., Lee W.M., Bonkovsky H.L. (2011). Maintenance peginterferon therapy and other factors associated with hepatocellular carcinoma in patients with advanced hepatitis C. Gastroenterology.

[15] Fattovich G., Giustina G., Degos F., Diodati G., Tremolada F., Nevens F., Almasio P., Solinas A., Brouwer J.T. (1997). Effectiveness of interferon α on incidence of hepatocellular carcinoma and decompensation in cirrosi type C. J. Hepatol..

[16] Cardoso A.C., Rami M.R., Figueiredo-Mendes C., Ripault M.P., Giuily N., Castelnau C., Boyer N., Tarik A.T., Martinot-Peignoux M., Maylin S. (2010). Impact of peginterferon and ribavirin therapy on hepatocellular carcinoma: Incidence and survival in hepatitis C patients with advanced fibrosis. J. Hepatol..

[17] Shiratori Y., Ito Y., Yokosuka O., Imazeki F., Nakata R., Tanaka N., Arakawa Y., Hashimoto E., Hirota K., Yoshida H. (2005). Antiviral therapy for cirrhotic hepatitis C: association with reduced hepatocellular carcinoma development and improved survival. Ann. Intern. Med..

[18] European Association for the Study of the Liver (2015). EASL Recommendations on Treatment of Hepatitis C 2015. J. Hepatol..

[19] Charlton M., Everson G.T., Flamm S.L., Kumar P., Landis C., Brown R.S., Fried M.W., Terrault N.A., O'Leary J.G., Vargas H.E. (2015). Ledipasvir and Sofosbuvir Plus Ribavirin for Treatment of HCV Infection in Patients with Advanced Liver DiseaseSOLAR-1 Investigators. Gastroenterology.

[20] Bunchorntavakul C., Reddy K.R. (2015). Review article: The efficacy and safety of daclatasvir in the treatment of chronic hepatitis C virus infection. Aliment. Pharmacol. Ther..

[21] Afdhal N., Zeuzem S., Kwo P., Chojkier M., Gitlin N., Puoti M., Romero-Gomez M., Zarski J.P., Agarwal K., Buggisch P., ION-1 Investigators (2014). Ledipasvir and sofosbuvir for untreated HCV genotype 1 infection. N. Engl. J. Med..

[22] Papatheodoridis G.V., Lampertico P., Manolakopoulos S., Lok A. (2010). Incidence of hepatocellular carcinoma in chronic hepatitis B patients receiving nucleos(t)ide therapy: A systematic review. J. Hepatol..

[23] Papatheodoridis G.V., Manolakopoulos S., Touloumi G., Vourli G., Raptopoulou-Gigi M., Vafiadis-Zoumbouli I., Vasiliadis T., Mimidis K., Gogos C., Ketikoglou I. (2011). Virological suppression does not prevent the development of hepatocellular carcinoma in HBeAg-negative chronic hepatitis B patients with cirrhosis receiving oral antiviral(s) starting with lamivudine monotherapy: Results of the nationwide HEPNET. Greece cohort study. Gut.

[24] Chang K.C., Hung C.H., Lu S.N., Wang J.H., Lee C.M., Chen C.H., Yen M.F., Lin S.C., Yen Y.H., Tsai M.C. (2012). A novel predictive score for hepatocellular carcinoma development in patients with chronic hepatitis C after sustained response to pegylated interferon and ribavirin combination therapy. J. Antimicrob. Chemother..

[25] Van der Meer A., Feld J., Hofer H., Almasio P., Calvaruso V., Fernandez-Rodriguez C., Aleman S., Ganne-Carrie N., D’Ambrosio R. (2013). The risk for hepatocellular carcinoma among patients with chronic HCV infection and advanced hepatic fibrosis following sustained virological response. Hepatology.

[26] Huang C.F., Yeh M.L., Tsai P.C., Hsieh M.H., Yang H.L., Hsieh M.Y., Yang J.F., Lin Z.Y., Chen S.C., Wang L.Y. (2014). Baseline gamma-glutamyl transferase levels strongly correlate with hepatocellular carcinoma development in non-cirrhotic patients with successful hepatitis C virus eradication. J. Hepatol..

[27] D'Ambrosio R., Aghemo A., Rumi M.G., Ronchi G., Donato M.F., Paradis V., Colombo M., Bedossa P. (2012). A morphometric and immunohistochemical study to assess the benefit of a sustained virological response in hepatitis C virus patients with cirrhosis. Hepatology.

[28] Maylin S., Martinot-Peignoux M., Moucari R., Boyer N., Ripault M.P., Cazals-Hatem D., Giuily N., Castelnau C., Cardoso A.C., Asselah T. (2008). Eradication of hepatitis C virus in patients successfully treated for chronic hepatitis C. Gastroenterology.

[29] Calle E.E., Rodriguez C., Walker-Thurmond K., Thun M.J. (2003). Overweight, obesity, and mortality from cancer in a prospectively studied cohort of U.S. adults. N .Engl. J. Med..

[30] Koh W.P., Robien K., Wang R., Govindarajan S., Yuan J.M., Yu M.C. (2011). Smoking as an independent risk factor for hepatocellular carcinoma: the Singapore Chinese Health Study. Br. J. Cancer.

[31] Baffy G., Brunt E.M., Caldwel S.H. (2012). Hepatocellular carcinoma in non-alcoholic fatty liver disease, an emerging menace. J. Hepatol..

[32] El-Serag H.B., Mason A.C. (2000). Risk factors for the rising rates of primary liver cancer in the United States. Arch. Intern. Med..

[33] Hassan M.M., Hwang L.Y., Hatten C.J., Swaim M., Li D., Abbruzzese J.L., Beasley P., Patt Y.Z. (2002). Risk Factors for Hepatocellular Carcinoma, Synergism of Alcohol With Viral Hepatitis and Diabetes Mellitus. Hepatology.

[34] Allen E., Beral V., Casabonne D., Kan S.W., Reeves G.K., Brown A., Green J., Million Women Study Collaborators (2009). Moderate alcohol intake and cancer incidence in women. JNCI.

[35] Baan R., Straif K., Grosse Y., Secretan B., El Ghissassi F., Bouvard V., Altieri A., Cogliano V., WHO International Agency for Research on Cancer Monograph Working Group (2007). Carcinogenicity of alcoholic beverages. Lancet Oncol..

[36] Lieber C.S., Seitz H.K., Garro A.J., Worner T.M. (1979). Alcohol-related diseases and carcinogenesis. Cancer Res..

[37] Lieber C.S. (1990). Mechanism of ethanol induced hepatic injury. Pharmacol. Ther..

[38] Iwasaki Y., Takaguchi K., Ikeda H., Makino Y., Araki Y., Ando M., Kobashi H., Kobatake T., Tanaka R., Tomita M. (2004). Risk factors for hepatocellular carcinoma in Hepatitis C patients with sustained virologic response to interferon therapy. Liver Int..

[39] Tokita H., Fukui H., Tanaka A., Kamitsukasa H., Yagura M., Harada H., Okamoto H. (2005). Risk factors for the development of hepatocellular carcinoma among patients with chronic hepatitis C who achieved a sustained virological response to interferon therapy. J. Gastroenterol. Hepatol..

[40] Arase Y., Kobayashi M., Suzuki F., Suzuki Y., Kawamura Y., Akuta N., Kobayashi M., Sezaki H., Saito S., Hosaka T. (2013). Effect of type 2 diabetes on risk for malignancies includes hepatocellular carcinoma in chronic hepatitis C. Hepatology.

[41] Davila J.A., Morgan R.O., Shaib Y., McGlynn K.A., El-Serag H.B. (2005). Diabetes increases the risk of hepatocellular carcinoma in the United States, a population based case control study. Gut.

[42] Polesel J., Zucchetto A., Montella M., Dal Maso L., Crispo A., La Vecchia C., Serraino D., Franceschi S., Talamini R. (2009). The impact of obesity and diabetes mellitus on the risk of hepatocellular carcinoma. Ann. Oncol..

[43] Bianchini F., Kaaks R., Vainio H. (2002). Overweight, obesity, and cancer risk. Lancet Oncol..

[44] Chen C.L., Yang H.I., Yang W.S., Liu C.J., Chen P.J., You S.L., Wang L.Y., Sun C.A., Lu S.N., Chen D.S. (2008). Metabolic factors and risk of hepatocellular carcinoma by chronic hepatitis B/C infection, a follow-up study in Taiwan. Gastroenterology.

[45] Angulo P. (2002). Nonalcoholic fatty liver disease. N. Engl. J. Med..

[46] Dyson J., Jaques B., Chattopadyhay D., Lochan R., Graham J., Das D., Aslam T., Patanwala I., Gaggar S., Cole M. (2014). Hepatocellular cancer, The impact of obesity, type 2 diabetes and a multidisciplinary team. J. Hepatol..

[47] Guzman G., Brunt E.M., Petrovic L.M., Chejfec G., Layden T.J., Cotler S.J. (2008). Does nonalcoholic fatty liver disease predispose patients to hepatocellular carcinoma in the absence of cirrhosis?. Arch. Pathol. Lab. Med..

[48] Tanaka A., Uegaki S., Kurihara H., Aida K., Mikami M., Nagashima I., Shiga J., Takikawa H. (2007). Hepatic steatosis as a possible risk factor for the development of hepatocellular carcinoma after eradication of hepatitis C virus with antiviral therapy in patients with chronic hepatitis C. World J. Gastroenterol..

[49] El-Zayadi A.R. (2006). Heavy smoking and liver. World J. Gastroenterol..

[50] Wu W.K.K., Wong H.P.S., Yu L., Cho C.H., Cho C.H., Purohit V. (2006). Nicotine and Cancer, in Alcohol Tobacco and Cancer.

[51] Gandini S., Botteri E., Iodice S., Boniol M., Lowenfels A., Maisonneuve P., Boyle P. (2008). Tobacco smoking and cancer: a metaanalysis. Int. J. Cancer.

[52] Wang L.Y., You S.L., Lu S.N., Ho H.C., Wu M.H., Sun C.A., Yang H.I., Chien-Jen C. (2003). Risk of hepatocellular carcinoma and habits of alcohol drinking, betel quid chewing and cigarette smoking, a cohort of 2416 HBsAg seropositive and 9421 HBsAg-seronegative male residents in Taiwan. Cancer Causes Control.

[53] Mori M., Hara M., Wada I., Hara T., Yamamoto K., Honda M., Naramoto J. (2000). Prospective study of hepatitis B and C viral infections, cigarette smoking, alcohol consumption, and other factors associated with hepatocellular carcinoma risk in Japan. Am. J. Epidemiol..

[54] Chen Z.M., Liu B.Q., Boreham J., Wu Y.P., Chen J.S., Peto R. (2003). Smoking and Liver Cancer in China, Case-Control Comparison of 36,000 Liver Cancer Deaths *vs.* 17,000 Cirrhosis Deaths. Int. J. Cancer.

[55] Franceschi S., Montella M., Polesel J., La Vecchia C., Crispo A., dal Maso L., Casarin P., Izzo F., Tommasi L.G., Chemin I. (2006). Hepatitis viruses, alcohol, and tobacco in the etiology of hepatocellular carcinoma in Italy. Cancer Epidemiol. Biomark. Prev..

